# Abnormal shortened diastolic time length at increasing heart rates in patients with abnormal exercise-induced increase in pulmonary artery pressure

**DOI:** 10.1186/1476-7120-9-36

**Published:** 2011-11-21

**Authors:** Tonino Bombardini, Rosa Sicari, Elisabetta Bianchini, Eugenio Picano

**Affiliations:** 1 National Research Council, Institute of Clinical Physiology, Pisa, Italy

**Keywords:** Diastolic time, Pulmonary hypertension; Cutaneous heart sensor

## Abstract

**Background:**

The degree of pulmonary hypertension is not independently related to the severity of left ventricular systolic dysfunction but is frequently associated with diastolic filling abnormalities. The aim of this study was to assess diastolic times at increasing heart rates in normal and in patients with and without abnormal exercise-induced increase in pulmonary artery pressure (PASP). Methods. We enrolled 109 patients (78 males, age 62 ± 13 years) referred for exercise stress echocardiography and 16 controls. The PASP was derived from the tricuspid Doppler tracing. A cut-off value of PASP ≥ 50 mmHg at peak stress was considered as indicative of abnormal increase in PASP. Diastolic times and the diastolic/systolic time ratio were recorded by a precordial cutaneous force sensor based on a linear accelerometer.

**Results:**

At baseline, PASP was 30 ± 5 mmHg in patients and 25 ± 4 in controls. At peak stress the PASP was normal in 95 patients (Group 1); 14 patients (Group 2) showed an abnormal increase in PASP (from 35 ± 4 to 62 ± 12 mmHg; P < 0.01). At 100 bpm, an abnormal (< 1) diastolic/systolic time ratio was found in 0/16 (0%) controls, in 12/93 (13%) Group 1 and 7/14 (50%) Group 2 patients (p < 0.05 between groups).

**Conclusion:**

The first and second heart sound vibrations non-invasively monitored by a force sensor are useful for continuously assessing diastolic time during exercise. Exercise-induced abnormal PASP was associated with reduced diastolic time at heart rates beyond 100 beats per minute.

## Introduction

Pulmonary hypertension is frequent in patients with heart failure, contributes to exercise intolerance and is associated with a worse outcome [1]. The degree of pulmonary hypertension is not independently related to the severity of left ventricular systolic dysfunction but is frequently associated with left ventricular diastolic filling abnormalities and with the quantified degree of functional mitral regurgitation [2]. It has been shown that dynamic mitral regurgitation and limited contractile reserve correlate with pulmonary pressure at exercise [3]. However, no study related the diastolic time duration during exercise with the pulmonary pressure at exercise [4,5]. Ideally, the non-invasive, imaging-independent, objective assessment of diastolic and systolic times at rest and during stress in patients with abnormal exercise-induced increase in pulmonary artery pressure would greatly enhance its practical appeal [6]. A new cutaneous force-frequency relation recording system has recently been validated in the stress echo lab, based on heart sound amplitude and timing variations at increasing heart rates [7,8]. Expert monitoring of the heart - via a chest wall accelerometer - can reliably and non-invasively sense the contractile force and the filling function of the heart [7,8]. The aim of this study was to compare the sensor-based quantification of diastolic times and diastolic/systolic time ratios with standard echo assessment during exercise stress echo in normals and in unselected patients with and without abnormal exercise-induced increase in pulmonary artery pressure.

## Methods

### Patient selection

We enrolled 109 consecutive patients referred for exercise stress echocardiography (78 males, age 62 ± 13 years) and 16 volunteers as controls. The characteristics of the study patients are reported in Table 1. All of the patients gave their written informed consent before entering the study. Volunteer controls were selected from our departments of Cardiology amongst subjects investigated for non-competitive sports eligibility. None of the control subjects had structural or functional cardiovascular abnormalities.

**Table 1:** Clinical study population findings

	Abnormal exercise PASP	Normal exercise PASP	P =
N of pts	14	95	

Dilated cardiomyopathy	1 (7%)	8 (8.5%)	NS

Aortic stenosis, moderate	3 (21%)	4 (4.2%)	< 0.05

Aortic regurgitation, moderate	0	2 (2.1%)	NS

Mitral stenosis, moderate	1 (7%)	0	NS

Mitral regurgitation, moderate	4 (29%)	3 (3.2%)	< 0.05

Known coronary artery disease	3 (21%)	45 (47.4%)	< 0.05

Prior myocardial infarction	1 (7%)	35 (37.6%)	< 0.05

Prior percutaneous coronary intervention	2 (14%)	32 (33.7%)	NS

Arterial hypertension	7 (50%)	46 (48.4%)	NS

Chronic obstructive pulmonary disease	0	18 (18.9%)	< 0.05

Diabetes	1 (7%)	20 (22.7%)	NS

Effort dyspnea	10 (77%)	39 (41%)	< 0.05

Left bundle branch block	3 (21%)	12 (12.6%)	NS

Right bundle branch block	0	8 (8.5%)	NS

Therapy at the time of test			

β-blockers	7 (50%)	40 (42.1%)	NS

Calcium antagonists	3 (21%)	26 (27.4%)	NS

Nitrates	1 (7%)	17 (18.3%)	NS

ACE inhibitors	5 (36%)	34 (36.6%)	NS

Diuretics	4 (29%)	13 (13.7%)	NS

At least one medication	10 (71%)	69 (72.6%)	NS

Data presented number (%) of patients.

PASP, pulmonary artery systolic pressure

All patients met the following inclusion criteria: 1) referred to stress echo for clinically driven testing; 2) sinus rhythm; 3) negative stress echocardiography. Of the initially considered 144 patients, 35 were excluded for one of the following reasons: 1) severe valvular disease or congenital heart disease (n = 11); 2) unwillingness to enter the study (n = 2); 3) poor acoustic window (n = 4) or unfeasibility of the spectral profile of tricuspid regurgitation (n = 14); positive stress echo (n = 4). The study complies with the Declaration of Helsinki. Informed consent was obtained from all patients (or their guardians) before testing, and the study protocol was approved by the institutional ethics committee. Stress echo data were collected and analyzed by stress echo cardiographers not involved in patient care. Indications for stress echocardiography were suspected coronary artery disease in 40 (37%) and risk assessment of known coronary artery disease in 48 (44%) cases, prognostic stratification in idiopathic (4 cases) or ischemic dilated (5 cases) cardiomyopathy, valvular heart disease in 17 (moderate mitral regurgitation, n = 7; moderate mitral stenosis, n = 1; moderate aortic stenosis, n = 7; moderate aortic regurgitation, n = 2); 18 patients with suspected coronary artery disease had established chronic obstructive pulmonary disease with effort dyspnea. Known coronary artery disease was history of myocardial infarction or coronary revascularization and/or presence of ≥ 1 angiographically documented coronary stenosis > 50%. Stress echocardiography was performed on medical therapy in 79 (72%) patients (Table 1).

### Stress Protocol

Two-dimensional echocardiography and 12-lead ECG monitoring were performed, in combination with semisupine exercise stress in accordance with well-established protocols [9]. A symptom-limited graded bicycle exercise test was performed in the semi-supine (45°) position on a tilting exercise table, keeping the legs at the seat level. During the procedure, blood pressure and ECG were recorded every minute. Quality control of stress echo performance and reading in enrolled centers was previously described in depth [10].

### Echocardiographic analysis

Echocardiographic images were semi-quantitatively assessed using a 17-segment, four-point scale model of the left ventricle [9]. A wall motion score index was derived by dividing the sum of individual segment scores by the number of interpretable segments. Left ventricular ejection fraction was assessed using the biplane Simpson method [11]. Ischemia was defined as stress-induced new and/or worsening of pre-existing wall motion abnormality, or biphasic response (that is, low-load improvement followed by high-load deterioration).

### Color-Doppler analysis

Valvular regurgitation was semi-quantified from color Doppler imaging and categorized as absent, minimal (within normal limits), mild, moderate, or severe [12].

### Non-invasive pulmonary artery systolic pressure

The pulmonary artery systolic pressure was derived from the maximal velocity of tricuspid Doppler tracing according to the Bernoulli equation, and adding the value of the right atrial pressure [13]. Right atrial pressure (mmHg) was estimated from the inferior vena cava diameter adjacent to the right atrium in the subcostal view. A inferior vena cava diameter < 2.1 cm that collapsed > 50% with a sniff suggested a normal right atrium pressure of 3 mm Hg (range, 0-5 mm Hg), whereas an inferior vena cava diameter > 2.1 cm that collapsed < 50% with a sniff suggested a high right atrium pressure of 15 mm Hg (range, 10-20 mm Hg). In indeterminate cases in which the inferior vena cava diameter and collapse do not fit this paradigm (inferior vena cava diameter > 2.1 cm that collapses > 50% with a sniff; or inferior vena cava diameter < 2.1 cm that collapses > 50% with a sniff) an intermediate value of 8 mm Hg (range, 5-10 mm Hg) was used. PASP was estimated at baseline and at peak exercise [13]. Normal resting values are usually defined as a peak tricuspid regurgitation gradient of < 31.4 to 33.6 mm Hg or a peak systolic pressure of 35 or 36 mm Hg, assuming a right atrium pressure of 3 to 5 mm Hg. A cut-off value of PASP ≥ 50 mmHg at peak stress was considered as indicative of abnormal exercise-induced increase in pulmonary artery pressure.

### Volume analysis

Left ventricular end-diastolic volume and end-systolic volume were obtained from apical four-chamber and two-chamber view using the biplane Simpson method [11]. The end-diastolic volume and end-systolic volume were assessed at rest and at peak of stress and normalized by dividing it by body surface area. Only representative cycles with optimal endocardial visualization were measured and the average of three measurements was taken. The endocardial border was traced, excluding the papillary muscles. The frame with the smallest left ventricular cavity was considered to be the end-systolic frame.

### End-systolic pressure-volume assessment

In order to build the end-systolic pressure-volume relation, the force was determined at rest and at peak of stress as the ratio of the end-systolic pressure/end-systolic volume index (biplane Simpson rule/body surface area). The end-systolic pressure-volume relation was built offline [14].

### Arterial elastance and ventricular-arterial coupling

In all, ventricular arterial coupling was indexed by the ratio of left ventricular systolic elastance index (systolic pressure/end-systolic volume index) to arterial elastance (ratio of end-systolic pressure by stroke volume). Echocardiography (for end-systolic volume and stroke volume) and cuff sphygmomanometer (systolic pressure, multiplied × 0.90 to obtain end-systolic pressure) provided the raw measurements. Because stroke volume (and input impedance) varies directly with body size, arterial elastance was adjusted for body surface area to better reflect differences in arterial properties with age and between the genders adjusted for differences in body size [15,16].

### Stroke volume index and cardiac index

The stroke volume was calculated as: stroke volume (mL) = end-diastolic volume - end-systolic volume. Stroke volume was indexed by dividing it by body surface area.

The Cardiac Output (L/min) was calculated as: heart rate*stroke volume. Cardiac output was indexed by dividing it by body surface area. Cardiac Index (L/min/m^2^) standardizes cardiac output for body size.

### Diastolic and systolic time measurements by precordial cutaneous sensor

All the enrolled subjects, scheduled for exercise stress, had standard ECG, pressure and complete (both stress and recovery) sensor evaluation. The transcutaneous force sensor is based on a linear accelerometer of the LIS3 family (STMicroelectronics [Geneva, Switzerland]). The device includes in a single package a micro electro-mechanical system sensor that measures capacitance variation in response to movement or inclination and a factory trimmed interface chip that converts the capacitance variations into analog signals proportional to the motion. The device has a full scale of ± 2·g (g = 9.8 m/s^2^) with a resolution of 0.0005·g. We housed the device in a small case which was positioned in the mid-sternal precordial region and was fastened by a solid gel ECG electrode. The acceleration signal is acquired along with an ECG signal and transmitted to a laptop PC by wireless connection. The data are analyzed using software developed in Matlab (The MathWorks, Inc Natick, Massachusetts, USA). A peak detector algorithm synchronized with the ECG scans the first 150 ms following the R-wave to record first heart sound force vibrations and the 100 ms following the T-wave to record second heart sound force vibrations. These values are then filtered by a median filter, which averages the beat-to-beat variations in the signal and removes outliers caused by movement artefacts. Contractility quantification through the first heart sound amplitude recording has been previously demonstrated [7,8]. Apart from the first and the second heart sound amplitude (related to the isovolumic contraction force and to the isovolumic relaxation force) this recording system was utilized to quantify both cardiological systole and diastole duration. According to the physiological background, cardiological systole is demarcated by the interval between the first and the second heart sounds, lasting from the first heart sound to the closure of the aortic valve. The remainder of the cardiac cycle is automatically recorded as cardiological diastole [2]. All the parameters were acquired at baseline, during stress and recovery.

### Statistical analysis

Statistical Package for Social Science 11 for Windows was utilized for statistical analysis. The statistical analyses included descriptive statistics (frequency and percentage of categorical variables and mean and standard deviation of continuous variables). Pearson chi-square with Fisher's exact test for categorical variables and the Mann-Whitney test for continuous variables for inter-group comparisons were performed to confirm significance (using Monte Carlo method for small sample comparisons). One-way analysis of variance was used to compare continuous variables between groups; when homogeneity of variance was not present, the Kruskal-Wallis test for nonparametric independent samples was used. Intergroup comparison was performed with Scheffe and Tamhane post hoc tests, respectively. Relations between variables were assessed using linear regression analysis and Pearson's correlation coefficient. Analysis of variance with Fisher's post hoc pairwise multiple comparisons was used to assess the significance of intragroup repeated measures.

## Results

By selection, technically adequate images were obtained in all patients at baseline and during stress, and no test was interrupted because of limiting side effects. Resting left ventricular ejection fraction in the entire study population was 57 ± 12%.

### Characteristics: baseline and exercise

By selection, the 109 patients performed the exercise test without chest pain, ST-segment depression, or echocardiographic evidence of inducible ischemia. The hemodynamic and Doppler echocardiographic variables obtained during exercise are shown in Table 2. The 14 patients with abnormal exercise-induced increase in pulmonary artery pressure, despite similar rest values, had blunted left ventricular based and end-systolic pressure/end-systolic volume index based contractile reserve, and less effective ventricular arterial coupling changes vs controls; values were lower but not statistically significant vs patients without abnormal exercise-induced increase in PASP. The patients with abnormal exercise-induced PASP had at rest in 2 cases mild and in 4 cases moderate mitral regurgitation that increased in 1 case from mild to moderate and in 3 cases from moderate to severe during stress. Three patients had moderate aortic and 1 moderate mitral stenosis; 3 patients had coronary artery disease (2 patients with previous percutaneous coronary intervention, 1 patient with previous myocardial infarction); 1 patient had diabetic dilated cardiomyopathy.

**Table 2:** Rest and stress data

	Patients, abnormal exercise PASP	Patients, normal exercise PASP	Controls
N of patients	14	95	16

Age (years)	63 ± 12*	60 ± 12 Δ	35 ± 9

Gender (Male/Female)	8/6	70/25	14/2

**Rest and stress echo measurements**			

Left ventricular mass index (g/m^2^)	112 ± 17	107 ± 30	99 ± 24

Heart rate rest (bpm)	65 ± 15	73 ± 15	77 ± 15

Heart rate peak (bpm)	118 ± 22*	123 ± 21 Δ	163 ± 11

Systolic blood pressure rest (mmHg)	128 ± 21	135 ± 20	126 ± 13

Systolic blood pressure peak (mmHg)	177 ± 21	186 ± 24	192 ± 27

Diastolic blood pressure rest (mmHg)	70 ± 10	75 ± 12	72 ± 10

Diastolic blood pressure peak (mmHg)	88 ± 11	94 ± 12	89 ± 13

Pulmonary artery systolic pressure rest (mmHg)	35 ± 4§	30 ± 5 Δ	25 ± 4

Pulmonary artery systolic pressure peak (mmHg)	62 ± 12§	38 ± 7 Δ	32 ± 6

LV end-diastolic volume index rest (mL/m^2^)	55 ± 23	52 ± 18	51 ± 13

Δ LV end-diastolic volume index (rest-peak, mL/m^2^)	-5 ± 15	-5 ± 9	-10 ± 11

LV end-systolic volume index rest (mL/m^2^)	22 ± 10	22 ± 14	21 ± 9

Δ LV end-systolic volume index (rest-peak, mL/m^2^)	-2 ± 6*	-4 ± 7 Δ	-10 ± 5

LV ejection fraction % rest	57 ± 14	57 ± 11	60 ± 7

Δ LV ejection fraction % (rest-peak)	1 ± 8*	5 ± 11 Δ	13 ± 9

Wall motion score index rest	1.12 ± 0.32	1.19 ± 0.37 Δ	1.00 ± 0.00

Systolic pressure/End-systolic volume index rest (mmHg/mL/m2)	8.6 ± 7.8	8.3 ± 5.4	7.1 ± 3.1

Δ Systolic pressure/End-systolic volume index (rest-peak, mmHg/mL/m^2^)	4.2 ± 5*	7.4 ± 7.9 Δ	14.6 ± 11.1

Arterial elastance index rest (mmHg/mL/m^2^)	4.3 ± 1.7	4.6 ± 1.5	4 ± 0.9

Δ Arterial elastance index (rest-peak, mmHg/mL/m^2^)	1.5 ± 1.8	1.7 ± 1.6	2.4 ± 2

Ventricular/arterial coupling rest (ratio)	1.7 ± 0.8	1.6 ± 0.8	1.6 ± 0.5

Δ Ventricular/arterial coupling (rest-peak)	0.4 ± 1.1	0.7 ± 1.3	1.6 ± 1.8

Cardiac index rest (L/min/m^2^)	1.99 ± 0.74	2.01 ± 0.70	2.19 ± 0.51

Δ Cardiac index (rest-peak, L/min/m^2^)	1.36 ± 0.94*	1.44 ± 0.95 Δ	2.58 ± 1.77

**Sensor built Force-Frequency Relation**			

Force rest (g * 10^-3^)	12.6 ± 10	9.7 ± 5.3 Δ	16.6 ± 7.4

Δ Force (rest-peak, g * 10^-3^)	21.4 ± 21.7*	22.5 ± 21.4 Δ	53.1 ± 33

**Sensor recorded diastolic and systolic times**			

*Diastolic time at 80 bpm (msec)*	415 ± 30 §	438 ± 25 Δ	470 ± 29

Diastolic time at 100 bpm (msec)	304 ± 19 §	323 ± 21 Δ	350 ± 22

Diastolic time at 110 bpm (msec)	264 ± 16 §	286 ± 21 Δ	306 ± 21

*Systolic time at 80 bpm (msec)*	335 ± 30 §	312 ± 25 Δ	280 ± 29

Systolic time at 100 bpm (msec)	303 ± 19 §	276 ± 21 Δ	250 ± 22

Systolic time at 110 bpm (msec)	281 ± 16 §	259 ± 21 Δ	239 ± 21

*Diastolic/systolic time ratio at 80 bpm*	1.26 ± 0.20 §	1.42 ± 0.20 Δ	1.76 ± 0.34

Diastolic/systolic time ratio at 100 bpm	1.01 ± 0.12 §	1.18 ± 0.17 Δ	1.42 ± 0.22

Diastolic/systolic time ratio at 110 bpm	0.95 ± 0.11 §	1.12 ± 0.17 Δ	1.29 ± 0.20

Diastolic/systolic time ratio at recovery, 100 bpm	1.24 ± 0.28 *	1.48 ± 0.28 Δ	1.75 ± 0.41

§ = significant differences between abnormal stress PASP patients vs. both normal stress PASP patients and Controls; ‡ = significant differences between abnormal stress PASP patients vs. normal stress PASP patients; * = significant differences between abnormal stress PASP patients vs. Controls. Δ = significant differences between normal stress PASP patients vs. Controls.

*g *= 9.8 m/sec^2^; PASP, pulmonary artery systolic pressure

### Sensor diastolic and systolic time monitoring at increasing heart rates

**Figure 1:**
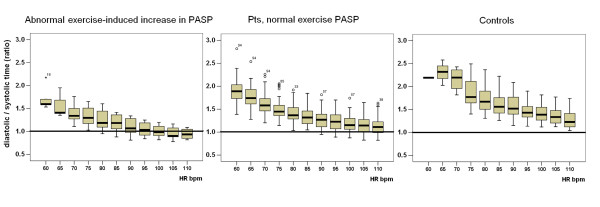
**Boxplots of diastolic/systolic time ratios at increasing heart rates (HR, bpm) in patients with (left panel) vs patients without (middle panel) exercise-induced increase in pulmonary artery systolic pressure**. Right panel, controls. At intermediate (100 bpm) heart rates an abnormal (< 1) diastolic/systolic time ratio was found in 0/16 (0%) controls, in 7/14 (50%) patients with and 12/93 (13%) patients without abnormal exercise-induced increase in pulmonary artery pressure, p < 0.05 between groups.

A consistent first heart sound and second heart sound signal was obtained in 107 out of 109 patients at rest and during stress. In 2 patients data were discarded because of a low signal-to-noise ratio which was related to both small amplitude of the signal and the presence of several artifacts. From rest to peak exercise, the mean systolic time was shortened by 23 ± 11%. The diastolic time decreased more markedly during exercise (by 50 ± 14% at peak stress). At 100 bpm heart rate during exercise, 19 patients showed a reversal of the diastolic/systolic ratio, with the duration of systole longer than that of diastole. The patients with abnormal exercise-induced increase in pulmonary artery pressure showed both at low (80 bpm) intermediate (100 bpm) and 110 bpm during stress prolonged systolic times, reduced diastolic times, and reduced diastolic/systolic time ratios. At intermediate (100 bpm) heart rates an abnormal (< 1) diastolic/systolic time ratio was found in 0/16 (0%) controls, in 7/14 (50%) patients with and 12/93 (13%) patients without abnormal exercise-induced increase in PASP, p < 0.05 between groups (Figure 1).

### Diastolic times in the post-exercise

The diastolic time increased abruptly during the first minute of recovery, with an overshoot phenomenon. At each recovery heart beat frequency, the diastolic time was higher than the diastolic time recorded during exercise, all p < 0.05 vs exercise. In the post-exercise period, diastole lengthened in both controls and patients: at recovery 100 bpm heart rate, + 36 ± 30 msec lengthening in controls vs + 23 ± 22 msec lengthening in patients with abnormal exercise-induced increase in PASP, vs + 33 ± 21 msec in patients with normal exercise PASP (p = ns between groups).

### Peak stress heart rate, diastolic time, and symptoms

**Figure 2:**
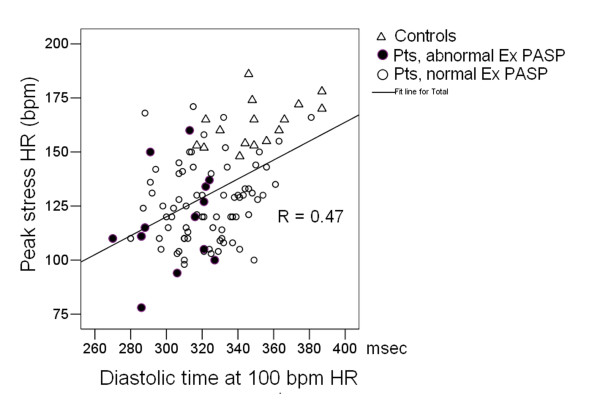
**Correlation between diastolic time (mesc) during exercise and peak stress HR (bpm) in controls (triangles), in patients with (full circles) and without (empty circles) abnormal exercise-induced increase in pulmonary artery systolic pressure**. The total cardiac cycle duration is algebraically dependent on the heart rate [= 60,000 msec/heart rate] with fixed values totally independent from the increasing heart rate stress type. At 100 bpm heart rate the cardiac cycle duration is = 600 msec. A longer diastolic time fraction improves ventricular filling and coronary perfusion time.

Peak stress heart rate was higher in controls vs patients with and without abnormal exercise induced increase in PASP (Table 2). Upper heart rate comparisons of diastolic times and diastolic/systolic time ratios were made at 110 bpm values: only 6/14 patients with, and 50/95 patients without exercise-induced increase in PASP, had peak stress heart rate ≥ 120 bpm. Exercise was symptom-limited by effort dyspnea in 10/14 patients with, and in 39/95 patients without abnormal exercise-induced increase in PASP, and in no control. A significant linear correlation (R = 0.47, p < 0.001) between diastolic time duration at 100 bpm heart rate and peak stress heart rate was found (Figure 2).

## Discussion

Increased systolic pulmonary artery pressure during exercise has been hypothesized to signify occult left ventricular dysfunction. In a previous study in a selected population of patients with chronic severe mitral regurgitation and apparently normal left ventricular function, Agricola [17] showed that the appearance of abnormal exercise-induced increase in PASP was related to the presence of left ventricular dysfunction, which appeared as a flat or biphasic Force-Frequency Relation. Tuminiello [3] showed that in patients with heart failure, dynamic mitral regurgitation and limited contractile reserve correlated with pulmonary pressure at exercise; the magnitude of pulmonary pressure during exercise in these patients mainly depends on dynamic mitral regurgitation. In another study, in a selected population of patients with Primary Pulmonary Hypertension, Marcus [18] showed that right ventricular pressure overload leads to leftward ventricular septal bowing and reduced right ventricular output. With decreased blood delivery, left ventricular filling is reduced, which results in decreased left ventricular stroke volume by the Frank-Starling mechanism.

In our study we found that abnormal exercise-induced PASP was associated with blunted left ventricular contractile reserve as assessed by the systolic pressure/end-systolic volume index relation. Furthermore, for the first time, we found an association with reduced diastolic time and with diastolic/systolic time mismatch at heart rates beyond 80 beats per minute.

During exercise, an increase in pulmonary artery pressure may theoretically result from a reduction in time available for the diastolic mitral inflow in the face of a fully distended circuit [19] and a rise in left atrial pressure.

### Operator-Independent sensor-measured diastolic time and diastolic/systolic time ratio during stress

First heart sound vibrations are an expression of the tension wave produced during initial activation of the heart in the isovolumic contraction phase and are fairly consistent in sinus rhythm. Second heart sound vibrations generates from the abrupt deceleration of the moving aortic blood mass at the beginning of the isovolumic relaxation phase [20]. We demonstrated in a minipig model of chronic heart failure that the force-frequency relation can be assessed noninvasively by a transthoracic sensor based on an accelerometer, with potential for wearable heart failure monitoring [21].

Feasibility in humans and reproducibility of the first cardiac tone, of the second cardiac tone amplitude monitoring, and of diastolic time assessment at increasing heart rates induced by exercise or pharmacological stressors has been previously validated [7,8,22]. In this study the sensor-monitored first and second heart sound vibrations were utilized as time markers to continuously assess cardiologic systole and diastole during exercise, in a non-invasive, imaging-independent, objective assessment. Heart rate is the major determinant affecting diastole and systole duration. Systole is linearly related to heart rate, with the ejection time inversely related to heart rate. Diastole has a more complex relation to heart rate and is longer at low heart rates [6]. The duration of diastasis, its shortening, and eventual disappearance as heart rate increases is the dominant factor accounting for the shortening of diastole duration. The total cardiac cycle duration is algebraically dependent on the heart rate [= 60,000 msec/heart rate]. However at each heart rate the fixed total cardiac cycle time can be differently divided between systole and diastole. At a normal heart rate of 75 bpm, the period of contraction constitutes ≈ 34% of the cardiac cycle (R-R interval). At 200 bpm, it increases to ≈ 53% of the cardiac cycle [23]. In the failing heart, or in one with blunted contractile reserve, the systolic shortening that takes place as the heart rate increases is more limited compared with the normal heart. Consequently, the frequency at which hemodynamic efficiency is lost is decidedly lower. Prior work concerning diastolic duration has been motivated primarily by consideration of diastolic myocardial perfusion time rather than the duration of mechanical events and has been used to assess the heart rate-dependent effects of pharmacological agents [24-26]. Recent studies utilizing both exercise radionuclide angiography time activity curve [4] or Doppler echocardiography [5,6] assessed that cardiac performance may be characterized in terms of the relative duration of systole and diastole. Cardiac cycle abnormalities of patients with heart failure are characterized by a prolongation of left ventricular systole and an abnormal shortening of left ventricular diastole. Reversal of the normal diastolic/systolic ratio may compromise cardiac filling and function. The diastolic-systolic mismatch is accentuated during exercise and has the potential to impair the cardiac reserve in these patients by restricting ventricular filling and perfusion. The sensor-measured non-invasive, imaging-independent, objective assessment of diastolic and systolic times at rest and during stress would greatly enhance its practical appeal.

### Limitations

Several limitations should be acknowledged. We did not perform invasive measurement of either PASP at rest or during exercise, excluding assessment of pulmonary vascular resistance as an indicator of input impedance [27]. We used a similar estimation of right atrial pressure at rest and at exercise, thus missing the potential influence of exercise-induced increase in right atrial pressure. Second, our calculations of stroke volume and left-ventricular volumes were based on geometric assumptions, which although validated and accurate, are less precise than those obtained by cardiac magnetic resonance imaging, the current gold standard for volumetric left-ventricular analysis [28]. An alternative approach would be to use the aortic Doppler velocity-time integral at peak exercise as a surrogate for stroke volume, a technique which also has significant limitations [29]. A third potential limitation of this study is the lack of information regarding Doppler indexes or diastolic function or dysfunction during stress. During exercise E- and A-waves become difficult to separate and discern when the A-wave merges with the E-wave and covers more than two-thirds of the E-wave deceleration, which typically occurs at heart rate ≥ 100 beats/min [6]. A fourth potential limitation is the fact that cardiologic diastole includes filling phases, but also isovolumic relaxation [2]. Its inclusion may have a slight effect on our measured fitting parameters. The inclusion of isovolumic relaxation time, which is minimally heart rate-dependent, would essentially add a constant (shift) to the data. Because it is essentially a constant offset, it would not change the significant finding that it is the change in heart rate that modulates the normal or abnormal change in the duration of diastole.

### Clinical implications and different scenarios for use of the sensor in the stress echo lab

Stress-induced "diastolic-systolic mismatch" can be easily quantified by a disproportionate decrease of diastolic time fraction, and is associated to several cardiac diseases, possibly expanding the spectrum of information obtainable during stress echo. Doppler echocardiography is the standard method for non-invasive diastolic function assessment However there are technical difficulties in assessing Doppler variables that influence cardiac diastolic filling during exercise. Interpretable E/E' are not always obtained, since the most common source of uninterpretable tracings is fusion of E and A velocities due to tachycardia [6]. A better (more feasible and sensitive) way to assess diastolic function during stress echo is needed. The combination of a cutaneous operator-independent force sensor and stress echo allows a highly feasible, fast and informative assessment of the normal or abnormal diastolic/systolic time ratio that compromise cardiac filling and function. Furthermore the sensor-derived information is available up to 200 beats per minute, without the heart rate limits of diastolic Doppler indexes [8]. This may have clinical implications for patients with coronary artery disease, since coronary flow in mostly diastolic. At a given coronary perfusion pressure, subendocardial perfusion is dependent on the ratio between the time the heart is in diastole and the duration of a complete heart cycle. The diastolic time fraction indicates the duration of absence of compression of intramural vessels during a heart beat and is used as input into theoretical models on coronary perfusion [26,30]. The absence of a unique relation between heart rate and diastolic time fraction on one hand and the dominant role of diastolic time fraction in subendocardial perfusion on the other hand also follow from the observation that, at the ischemic threshold, diastolic time fraction rather than heart rate correlates with the significance of coronary stenosis in patients. This indicates the relevance of monitoring by the sensor the diastole time in the diseased heart.

### Future developments

Portability of the sensor and simple remote transmission of the signal could allow telemonitoring in chronic heart failure patients. Dichotomy patterns: upsloping vs flat systolic Force-Frequency Relation [7], stress-induced "diastolic/systolic mismatch (yes/no), are easily recognized by sensor-based intelligent monitoring. Our research will continue to optimize features of both the sensor and algorithm, and will develop an engineering model for industrialization, aiming at the device's eventual use in long-term home monitoring for tailoring drug treatment and preventing re-hospitalization [31].

## Conclusions

The sensor-monitored first and second heart sound vibrations were utilized as time markers to continuously assess diastolic time during exercise, in a non-invasive, imaging-independent, objective assessment. Abnormal exercise-induced PASP was associated with reduced diastolic time and with diastolic/systolic time mismatch at heart rates above 80 beats per minute. A blunted diastolic time fraction worsens ventricular filling and coronary perfusion time.

## Competing interests

The sensor for the diastolic time measurement is an embodiment of the United States Patent 6,859,662. Inventor: T.B.

## Authors' contributions

All authors read and approved the final manuscript. TB conceived and designed the research, performed statistical analysis and drafted the manuscript; EB analyzed the data; RS and EP did critical revision of the manuscript for important intellectual content.
